# Identification of differentially expressed subnetworks based on multivariate ANOVA

**DOI:** 10.1186/1471-2105-10-128

**Published:** 2009-04-30

**Authors:** Taeyoung Hwang, Taesung Park

**Affiliations:** 1 Interdisciplinary Program of Bioinformatics, Seoul National University, Seoul, Republic of Korea; 2 Department of Statistics, Seoul National University, Seoul, Republic of Korea

## Abstract

**Background:**

Since high-throughput protein-protein interaction (PPI) data has recently become available for humans, there has been a growing interest in combining PPI data with other genome-wide data. In particular, the identification of phenotype-related PPI subnetworks using gene expression data has been of great concern. Successful integration for the identification of significant subnetworks requires the use of a search algorithm with a proper scoring method. Here we propose a multivariate analysis of variance (MANOVA)-based scoring method with a greedy search for identifying differentially expressed PPI subnetworks.

**Results:**

Given the MANOVA-based scoring method, we performed a greedy search to identify the subnetworks with the maximum scores in the PPI network. Our approach was successfully applied to human microarray datasets. Each identified subnetwork was annotated with the Gene Ontology (GO) term, resulting in the phenotype-related functional pathway or complex. We also compared these results with those of other scoring methods such as *t *statistic- and mutual information-based scoring methods. The MANOVA-based method produced subnetworks with a larger number of proteins than the other methods. Furthermore, the subnetworks identified by the MANOVA-based method tended to consist of highly correlated proteins.

**Conclusion:**

This article proposes a MANOVA-based scoring method to combine PPI data with expression data using a greedy search. This method is recommended for the highly sensitive detection of large subnetworks.

## Background

Since the advent of microarray technology, genome-wide expression analysis has become an important tool in biology [1]. Much of the initial research with expression data has focused on evaluating the significance of individual genes in a comparison between two groups of samples to identify differentially expressed genes (DEGs). Various statistical approaches have been proposed in the literature [2-4]. However, the main difficulty lies not in the identification of DEGs but in their interpretation [5]. The interpretation of a long list of DEGs can be a daunting and ad hoc endeavor, since it is dependent on the biologist's area of expertise [6]. A compromise can be reached by constructing sets of individual genes (hereafter referred to as "gene sets") from prior biological data. For example, gene sets from a public database such as Gene Ontology (GO) and KEGG would provide functional interpretations of biological modules. In addition, by extending the level of analysis from an individual gene to multiple genes, we can identify genes with small changes that are not identified at the single-gene level analysis [7]. Furthermore, this multiple-gene approach is reasonable from a biological perspective because many genes function in concert rather than alone [8]. The hurdle, however, is that the majority of human genes have not yet been assigned to a definite pathway or complex. As protein-protein interaction (PPI) data become available, integrating PPI data with genome-wide expression data will provide new opportunities to at least partially address this challenge [9]. The combined analysis of expression data and PPI data allows us to detect unknown gene sets–those not obtainable from a public database [10]. Therefore, researchers could utilize this approach to formulate a novel hypothesis about a pathway.

The integration of PPI data with gene expression data requires an adequate scoring method to measure the discriminative potential of a given subnetwork or a connected subgraph. Several scoring methods have previously been proposed: combining multiple *p*-values [11], mutual information [9], and edge scoring [12,13]. In addition, it is possible to utilize protein structure information in the scoring method. For example, the fact that no two partners can interact with a given binding site at the same time provides data that is complementary to the gene expression data. In this way, the prediction of protein-protein interactions by utilizing the information of protein-protein interfaces in public databases provides additional information for proper scoring [14-17]. In addition to a proper scoring method, an effective search algorithm is required to find the subnetworks with the maximum scores. Various search algorithms have been suggested. For example, simulated annealing [13], greedy search [8,9], and exact search [10] have been proposed as search algorithms. In particular, Chuang et al. [9] used a greedy search with mutual information-based scoring method to classify breast cancer metastasis. They successfully identified the subnetworks related to breast cancer metastasis and showed the usefulness of integrated analysis for identifying a disease marker.

This paper focuses on a scoring method adopting when using a greedy search. It has been reported that while the correlation among the genes in a subnetwork is not conspicuous, it is important in assessing statistical significance [5]. The multivariate analysis of variance (MANOVA) model is a well-known statistical model used to determine whether significant mean differences exist among groups. One advantage of MANOVA is that the correlation structure is taken into consideration. Motivated by this characteristic feature of MANOVA, we propose a MANOVA-based scoring method for identifying differentially expressed PPI subnetworks. We also suggest several criteria appropriate for the comparison of scoring methods in the context of a greedy search algorithm.

## Results

### Biological significance of identified subnetworks

#### Fibroblast serum response

A greedy search with our MANOVA-based scoring function identified 86 significant subnetworks with *p *< 0.05. We performed the GO term enrichment analysis to investigate how well the identified subnetworks represented the functional modules. Under the assumption of a hyper-geometric distribution, we calculated the significance level for the number of proteins in an identified subnetwork that were included in each GO term in the category of "biological process" and selected most significant GO terms for functional annotations of the identified subnetwork. The ten most significant subnetworks are listed in Table 1. As shown in Table 1, the wound healing-relevant biological processes in GO, such as "cell-cell adhesion" (GO: 0016337), "fibroblast growth factor receptor signaling pathway" (GO: 0008543), and "blood coagulation, extrinsic pathway" (GO: 0007598), were enriched in our subnetworks. These are known as major biological processes related to wound healing.

**Table 1:** GO functional annotations for the ten most significant subnetworks (Serum Response data).

Sub-networks	GO annotation	P-value (GO)
**F10 F7 TFPI PRSS3 THBS1 FN1 MMP2 LTBP1 SDC2 CD36 F2 SERPING1 C1S**	**blood coagulation, extrinsic pathway**	**0**
**FGF2 FGFR1 CD44 EPB41L3 FGF7 FNDC5 EGFR DEGS1 FNDC4**	**fibroblast growth factor receptor signaling pathway**	**0**
GNB1 GNG4 GNG2 GNAS SNX13 ADCY9	hormone-mediated signaling	0
MCM7 MCM4 UBE3A MCM6 UBQLN2	DNA replication initiation	0
MAP2K1 ARAF RPS6KA2 ASS MAPK1 KLF11 NEK2 MBP PRKCD	protein amino acid phosphorylation	0
CEBPA CDK2 ATF2 MAPK11 DUSP14 NR3C1 HNRPU BCL2 PCNA TGFB1I1 SMARCA2	regulation of DNA replication	0.000003
**COL4A2 CD93 CD44 FGF2 FGFR1 EPB41L3 FNDC5 EGFR DEGS1 FNDC4 KRT17 CAMLG**	**cell-cell adhesion**	**0.000003**
DYNLL1 DNMT1 NOS1 TXNL5 BDKRB2 PCNA MSH3 RFC1 DCC1 CDK2 PARD3 CALM2	regulation of DNA replication	0.000004
**SDC3 FGF2 FGFR1 FNDC5 COL6A2 COL1A1 ITGA2 BGN P4HB API5**	**fibroblast growth factor receptor signaling pathway**	**0.000109**
**CSPG2 CD44 FGF2 FGFR1 EPB41L3 COL1A1 P4HB FNDC5 COL6A2 FNDC4 SDC3 MMP2**	**fibroblast growth factor receptor signaling pathway**	**0.000159**

GO terms are enriched for the identified subnetworks. *P*-values are the results of GO term enrichment analyses. The well-known biological processes related to wound healing are denoted by bold-faced letters.

#### Prostate cancer progression

In this example, 123 significant subnetworks were identified by our method. The ten most significant subnetworks are shown in Table 2. The "BMP signaling pathway" (GO: 0030509) and "epithelial to mesenchymal transition" (GO: 0001837) were observed. These processes have been reported to be essential to prostate cancer progression, showing how our results are useful [18,19].

**Table 2:** GO functional annotations for the ten most significant subnetworks (Prostate Cancer Metastasis data).

Sub-networks	GO annotation	P-value (GO)
**ACVR1 SMAD5 SMAD4 TFE3 DACH1 SPTBN1 JUN BMP2 SKIL MGP**	**BMP signaling pathway**	**0**
**ACVR1B TGFB3 TGFB1 BGN ITGAV ACVR2A BMP7**	**epithelial to mesenchymal transition**	**0**
**ACVR2A BMP6 SMAD5 SMAD4 ACVR1 ACVR1B TGFB1**	**BMP signaling pathway**	**0**
AKT2 PIK3R1 MME NRAS PIP5K2A TUBG1 NFKB1 TRIP4 PIK3C2B VAV2 CEBPB PIK4CA EGR1	phosphoinositide phosphorylation	0
ARF1 TMED2 TMED10 ARFGAP1 AP1G1 AFTIPHILIN PSCD2	vesicle-mediated transport	0
**BMP2 TGFB1 ITGAV BGN GLI2 LTBP1 TGFB3 BMP7 CSNK1A1 PRKY**	**epithelial to mesenchymal transition**	**0**
**BMP6 SMAD5 SMAD4 ACVR1 ACVR2A ACVR1B NCOA3 MYC GTF2B PCAF SMAD2**	**BMP signaling pathway**	**0**
**BMP7 ACVR1 SMAD5 SMAD4 ACVR2A ACVR1B TGFB1 BMP6 BMP2 TGFB3**	**epithelial to mesenchymal transition**	**0**
CANX MBTPS1 ITGB1 CD46 LGALS3BP ITGAV ITGB1BP1 PXN SERP1 ITGA8	cell-matrix adhesion	0
CAV1 PTPRF JUP ITGB4 COL17A1 NID1 LGALS3BP IRS1 FRS2 EDG1 PLEC1 APP TGFB1 CALR APPBP1	cell adhesion	0

GO terms are enriched for identified sub-networks. *P*-values are the results of GO term enrichment analyses. The well-known biological processes related to prostate cancer metastasis are denoted by bold-faced letters.

### Comparison with other methods

#### Real data sets

We compared our MANOVA-based scoring method with two other methods. Nacu et al. [8] suggested a scoring method based on averaging gene expression levels (hereafter referred to as the TΣ-based scoring method). And Chuang et al. proposed the mutual information-based scoring method [9] (hereafter referred to as the MI-based scoring method). We evaluated the performance of these three methods and compared the number of significant subnetworks, the size of each significant subnetwork, and the percentage of proteins with a higher correlation coefficient for a given seed protein in the subnetworks. In the case of the prostate cancer metastasis data, we used just two phenotypes–benign epithelium and primary prostate cancer–because the TΣ – based scoring method can only handle data consisting of two phenotypes. First, the number of significant subnetworks was determined, as shown in Table 3. The MANOVA-based scoring method produced the smallest number of significant subnetworks among the three methods.

**Table 3:** The number of significant networks identified by each scoring method.

Dataset	The number of seed proteins	TΣ	Mutual Information	MANOVA
Serum Response	154	129	100	86
Prostate Cancer Metastasis	140	124	92	83

**Figure 1:**
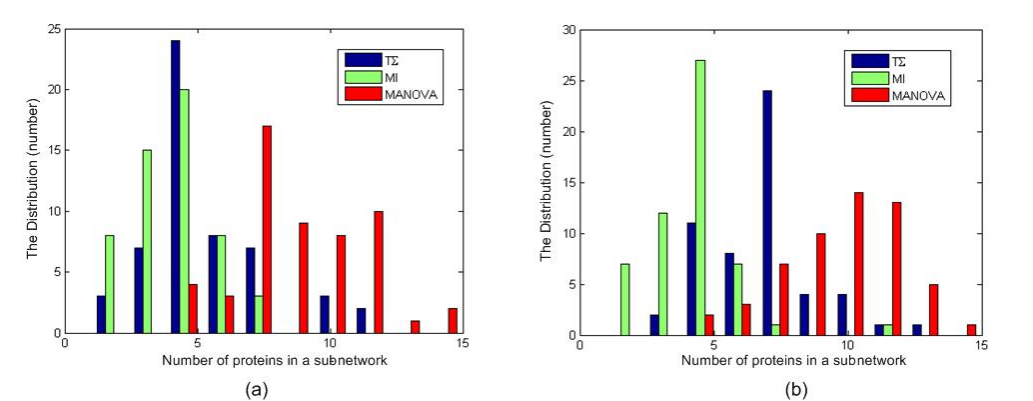
**The distribution of the number of proteins in a subnetwork**. (a) Serum response data. (b) Prostate cancer metastasis data.

Next, we examined the sizes of the significant subnetworks commonly identified by all three methods. Figure 1 shows the distribution of number of proteins in the commonly identified subnetworks. The MANOVA-based scoring method usually identified subnetworks with a larger number of nodes (genes) than the other methods. In summary, the MANOVA-based scoring method tended to yield a smaller number of significant subnetworks with larger numbers of proteins than the other scoring methods.

**Figure 2:**
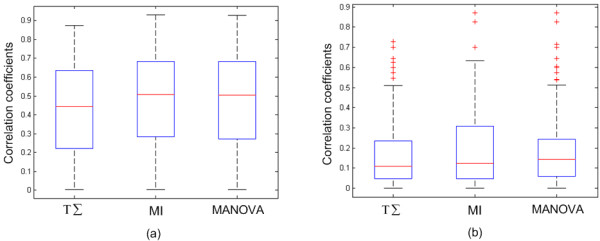
**The box plots of correlation coefficients between seeds and proteins in identified subnetworks**. (a) Serum response data. (b) Prostate cancer metastasis data. The correlation coefficients are absolute values.

Finally, we investigated the distribution of the correlation coefficients within the subnetworks identified as being significant by all three scoring methods to determine the strength of correlation among the proteins in the subnetworks. The correlation coefficient for every protein in the sub-networks with the seed protein was calculated. Figure 2 shows the distribution of the absolute values of the correlation coefficients for the subnetworks identified by each scoring method. The MI-based scoring method and the MANOVA-based scoring method had higher correlations than the TΣ – scoring method. We then calculated the percentages of the correlation coefficients higher than various thresholds. As shown by Tables 4 and Table 5, the MANOVA-based scoring method tended to have higher percentages than the other methods. This suggests that the MANOVA-based scoring method tends to construct subnetworks containing relatively larger numbers of highly co-regulated genes.

**Table 4:** The cumulative distribution of the number of proteins in the subnetworks (Serum response data).

correlation coefficient	TΣ	Mutual Information	MANOVA
≥ 0.5	36.14	41.28	**43.71**
≥ 0.6	20.08	25.1	**30.86**
≥ 0.7	10.14	**18.01**	17.87
≥ 0.8	2.49	2.93	**8.64**
≥ 0.9	0	0	0

The numbers denote the percentages of the number of proteins whose correlation coefficients are above the specific values in the subnetworks. The correlation coefficients are absolute values. The largest number in each row is denoted by bold-faced letters.

**Table 5:** The cumulative distribution of the number of proteins in the subnetworks (Prostate cancer data).

correlation coefficient	TΣ	Mutual Information	MANOVA
≥ 0.1	56.75	59.08	**62.5**
≥ 0.2	28.55	33.34	**36.25**
≥ 0.3	16.44	**26.35**	16.95
≥ 0.4	4.85	6.56	**7.78**
≥ 0.5	1.39	1.35	**1.81**
≥ 0.6	0.7	**0.9**	0.84

The numbers denote the percentages of the numbers of proteins whose correlation coefficients are above the specific values in the subnetworks. The correlation coefficients are absolute values. The largest number in each row is denoted by bold-faced letters.

#### Simulation study

**Figure 3:**
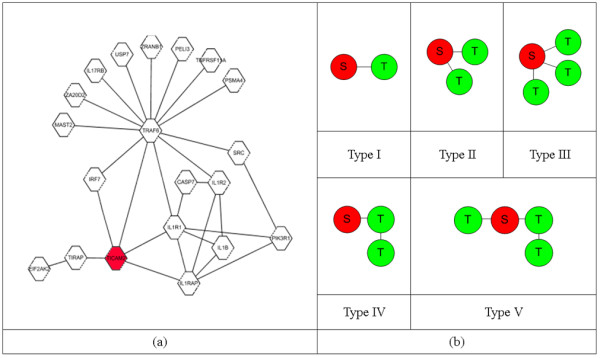
**The structures of the PPI network for the simulation study**. (a) An overall PPI network for the simulation study. (b) Five types of target subnetworks. The red nodes represent the seeds. The five types of network structures in (b) are assumed to be target networks for the PPI network (a).

We performed a series of simulation studies to evaluate the three scoring methods. Specifically, we focused on the ability of each method to identify previously assumed target subnetworks. The simulation study was performed for the PPI network in Figure 3a, which has proteins within a depth of two from a given seed protein. For this PPI network, five types of network structures were assumed to be true target networks (Figure 3b). Expression values with correlation coefficient *ρ *were assigned to the nodes of the target networks, while expression values with correlation coefficient *ρ' *were assigned to the other remaining nodes. The expression values with correlation coefficient *ρ *and *ρ' *were generated by the following procedure.

For the *k*^*th *^subnetwork, assume that there are *p*_*k *_nodes (genes) among which *t*_*k *_nodes (genes) belong to a given target network. For the *i*^*th *^gene in the phenotype group *g*, let *x*_*ig *_(*i *= 1, ⋯, *t*_*k*_; *g *= 1, ⋯, *G*) epresent the target node and y_*ig *_(*i *= 1, ⋯, *p*_*k*_; *g *= 1, ⋯, *G*) the nontarget node. Then, the expression values are generated to obtain a correlation coefficient of *ρ *for every pair in the target nodes and *ρ' *for every pair in the nontarget nodes by the following formulas:



where the *Z*s are normally distributed random variables and the *c *and *c' *are constants satisfying



For given values of *ρ *and *ρ'*, we calculated *c *and *c' *by setting *c*_0 _and  to be 0.001. Finally, we added the constant value *α *to the target nodes to make an average difference between the target nodes and nontarget nodes. *α *was assigned a value of 0.01. A greedy search was performed for each scoring method. Permutation tests were omitted in the simulation study because the expression values were generated from the normal distribution. If the identified subnetwork included all of the nodes of the target subnetworks, we considered it to be a successful identification. This procedure was repeated 100 times and the true positive (TP) rate was estimated as the number of successful identifications divided by 100. In order to estimate the false positive (FP) rate, every node (gene) in a subnetwork, regardless of whether it was a target or nontarget node, was assigned expression values of the same correlation coefficient value (*ρ *= *ρ' *in the above formulas).

We performed the simulation study with two cases of *ρ *and *ρ' *(0.8, 0.4; 0.8, 0.1) as shown in Table 6 and Table 7. The MANOVA-based scoring method had the highest sensitivity (TP rate) in both cases. In the case of specificity (true negative rate), the MI-based scoring method had the highest specificity.

**Table 6:** Sensitivity and specificity of each method in the simulation study (*ρ *= 0.8, *ρ' *= 0.4).

	Sensitivity			Specificity		
	TΣ	Mutual Information	MANOVA	TΣ	Mutual Information	MANOVA

Type I	0.36	0.27	0.56	0.66	0.69	0.5
Type II	0.07	0.04	0.18	0.9	0.94	0.79
Type III	0.01	0.01	0.07	0.98	1	0.87
Type IV	0.08	0.02	0.15	0.92	0.97	0.82
Type V	0.02	0.01	0.1	0.98	1	0.92

**Table 7:** Sensitivity and specificity of each method in the simulation study (*ρ *= 0.8, *ρ' *= 0.1).

	Sensitivity			Specificity		
	TΣ	Mutual Information	MANOVA	TΣ	Mutual Information	MANOVA

Type I	0.41	0.41	0.57	0.6	0.78	0.47
Type II	0.07	0.02	0.14	0.83	0.96	0.76
Type III	0.02	0.01	0.09	0.98	0.97	0.91
Type IV	0.08	0	0.24	0.87	0.98	0.77
Type V	0.02	0.01	0.04	0.94	1	0.92

## Discussion

This article proposes a MANOVA-based scoring method to identify subnetworks in a PPI network. Our method was successfully applied to two human microarray data sets, resulting in biologically significant PPI subnetworks. We also compared the results with those of two other scoring methods using a greedy search algorithm. The characteristics of these scoring methods when using a greedy search are not well known, because applying the different scoring methods to a greedy search algorithm usually generates subnetworks with different proteins and sizes. To our knowledge, our research is the first comparison study to evaluate the performance of various scoring methods in the context of a greedy search. We believe that our results provide a guide to develop and expedite the integration of PPI data with other biological data using a greedy search.

In an individual gene-level analysis [1-4], a list of DEGs is identified and examined for the enrichment of gene sets or subnetworks. In this case, DEGs are identified under the assumption of independence between genes. However, as several studies have pointed out, the correlation structure in a subnetwork is not a trivial problem in assessing the statistical significance of subnetworks [5,8]. MANOVA takes into account the correlation between multiple dependent variables when testing whether significant differences exist among groups. Our empirical studies showed this consistently. The MANOVA-based scoring method provides the smallest number of significant subnetworks. However, it tends to provide larger subnetworks with higher correlated proteins than the TΣ – based scoring method, which does not consider the correlation among genes. In addition, the simulation study showed that the MANOVA-based scoring method has a high sensitivity for the identification of true target-networks consisting of higher correlated proteins, though at the cost of low specificity. These characteristics could be emphasized further considering the report that hub proteins have a tendency to exhibit low expression values even though they are more essential [20]. Our method could detect significant subnetworks that have essential proteins with moderate expression levels around hub proteins by reflecting the correlation in the construction of the subnetworks, providing more opportunities to identify novel pathways or complexes around hub proteins than other methods.

Furthermore, the MANOVA-based scoring method can deal with the data of any number of groups. Our results show the successful identification of the subnetworks differentiating three groups related to prostate cancer metastasis, as well as two groups related to the wound response of a fibroblast culture. Since it is common for researchers to compare more than two experimental groups defined by multiple experimental factors, our method could provide a useful tool for identifying subnetworks under multiple conditions.

However, there are some assumptions when using the MANOVA approach [21]. First, it assumes a normal distribution of the dependent variables. However, we adopted a permutation test to derive *p*-values. Thus, the violation of this normal assumption is not critical to our method. Second, the correlation among the dependent variables is linear. Note that linear correlation has been successfully applied to the analysis of gene expression data in many previous studies [21,22]. Therefore, the assumption of linear correlation in MANOVA is biologically acceptable. Third, the variances and covariances of the dependent variables should be homogenous across the phenotype groups. Several tests may be applied to check the validity of this assumption. When this homogenous assumption is not satisfied, the transformation of the dependent variables may be recommended.

Our method adopted the greedy search for identifying PPI subnetworks. In contrast to approaches using a predefined gene set [5-7], greedy search has the potential to identify novel subnetworks. However, one shortcoming is the difficulty in creating a biological interpretation of the identified subnetworks. Moreover, the greedy search algorithm is not guaranteed to identify the highest scoring subnetwork because it is fundamentally a heuristic algorithm. To solve this problem, an algorithm for finding optimal-scoring subnetworks has recently been suggested [10]. However, methods of this kind generally involve huge computational complexity.

Finally, it is worth noting that other types of additional information could be used to improve our method. For example, managing multiple microarray experiments with annotations of the experimental conditions [23] would produce finer phenotype-related subnetworks. In addition, integrating other factors influencing the protein abundance, such as the miRNA activity and degradation rate of proteins, with the current algorithm could improve the performance of our method, because the gene expression level does not necessarily represent the true protein abundance.

## Conclusion

A MANOVA-based scoring method with a greedy search was proposed for combining PPI data with expression data. Our method takes advantage of a characteristic feature of MANOVA: it considers the correlation structure of multiple dependent variables when comparing the mean vectors across different groups. Our method was successfully applied to two human microarray data sets. The results from comparisons with two other scoring methods showed that the MANOVA-based scoring method tends to yield a smaller number of significant subnetworks with larger numbers of highly coregulated proteins. It also performed better in terms of sensitivity than other methods in simulation studies. Therefore, the MANOVA-based scoring method could provide more opportunities to identify novel pathways or complexes, including unknown genes or proteins related to the phenotypes of microarray experiments.

## Methods

Following the idea of Ideker et al. [11], we matched the expression values of each gene with its corresponding protein in the PPI network. We then searched for the subnetworks with the maximum MANOVA-based scores. The significance of the subnetworks was determined by a permutation test.

### Data

#### Expression data

We tested our method using two real expression data sets. One was related to the serum effect on fibroblast cultures, and the other involved the progression of prostate cancer in 44 individuals.

#### Fibroblast serum response

Chang et al. [24] studied the relationship between tumor growth and wound recovery using a microarray experiment. *In vivo *cells encounter serum, the soluble fraction of coagulated blood, when they are injured. Serum promotes the biological processes involved in wound healing by fibroblasts. To characterize wound response, Chang et al. [24] investigated the effect of serum on the expression profiles of fibroblast cultures. The expression data were obtained from Nacu et al. [8].

#### Prostate cancer progression

This data set was a collection of cDNA microarray expression measurements from 22 samples of benign epithelium, 32 samples of primary prostate cancer, and 17 samples of metastatic prostate cancer. The progression was benign to prostate cancer (PCA) to metastasis [25]. The data were obtained from NCBI GEO [26], and the accession number was GSE6099.

#### Protein-protein interaction data

We exploited the interaction dataset used in Nacu et al. [8] to test our scoring method and compare it with other scoring methods. To obtain human PPI network data, Nacu et al. [8] used data from EntrezGene (December 2005) and 33 human pathways in KEGG (March 2006). After removing loops (nodes connected to themselves), a graph with 7180 nodes and 27,082 edges was obtained.

### Scoring methods

A subnetwork is defined as a gene set that induces a single connected component in the PPI network [9]. Given a set of genes, we need to compute a score that measures how much the set is differentially expressed. Nacu et al. [8] suggested a scoring method based on averaging gene expression levels (hereafter referred to as "TΣ"). TΣ first sums the expression levels for the genes in the subnetwork and then computes the *t *statistic. Chuang et al. [24] proposed a mutual information-based scoring method (hereafter referred to as "MI") between the activity vector over the samples and the phenotype. The activity vector of a given subnetwork is defined by averaging their gene-wise normalized expression values.

We propose using Wilks' *Λ *statistic as a MANOVA-based score for a given subnetwork. MANOVA is an extension of analysis of variance (ANOVA) that covers cases where there is more than one dependent variable. In our case, we treat each gene of a subnetwork as a dependent variable. Suppose there are *K *subnetworks and *G *phenotype groups. For the *k*^*th *^subnetwork, assume that there are *p*_*k *_nodes (genes). For the *i*^*th *^gene in group *g*, let  be the expected value of its expression value. Then the following hypotheses are of interest:



MANOVA considers the correlation structure of multiple dependent variables when it is used to compare the mean vectors across different groups. Because subnetworks representing complexes or pathways usually consist of multiple coexpressed genes, we propose using the MANOVA model to consider these genes simultaneously.

### Search algorithms

Given the scoring functions, a greedy search is performed to identify subnetworks within the PPI network. In the beginning, each candidate subnetwork has a single seed protein. In this study, proteins with more than five interactions were chosen as seed proteins. To expand the subnetwork from a seed protein, we first construct every possible subnetwork consisting of the seed and each of its neighboring proteins. After completing the score calculation for all of the possible subnetworks, we choose the neighboring proteins in the subnetworks with the maximum scores and include them as members of expanded subnetworks. This process is iterated until the termination conditions are met. Three termination criteria are used. First, the search stops when no addition of neighbor proteins increases the score over a specified relative improvement rate *r*, which is defined as the difference between the previous and current scores divided by the previous score. Second, the distance from the seed is adopted as another criterion. That is, only proteins within a specified distance *d *from the seed are added to an expanded subnetwork. Finally, the maximum possible number of proteins in a subnetwork is also used as a termination criterion to avoid the singular matrix conversion in the process of calculating Wilks' *Λ *statistic from MANOVA. When one of the three criteria is satisfied, the iteration stops. Given that the median distance between any two proteins in the human PPI network is five, we set *d *to be 2 to provide a sufficient number of neighbors to keep the search local. A value of 0.05 is chosen for the parameter *r *[9]. The maximum number of nodes (genes) in a subnetwork is set to one less than the number of samples in the smallest group.

### Computing significance levels

We proposed a permutation-based test to derive the significance levels. The permutation-based approach provided a reasonable estimate for the null distribution of the subnetwork scores, and allowed us to compute *p*-values. The iteration number for this permutation test was set to 1000.

## Competing interests

The authors declare that they have no competing interests.

## Authors' contributions

All of the authors participated in the design of the methods. TH implemented the algorithm and analyzed the results. TP coordinated the research. All of the authors have read and approved the manuscript.
